# Research progress of knee fibrosis after anterior cruciate ligament reconstruction

**DOI:** 10.3389/fphar.2024.1493155

**Published:** 2024-10-21

**Authors:** YangYang Liang, QingQing Zhang, YouFei Fan

**Affiliations:** ^1^ Department of Sports Trauma and Arthroscopic Surgery, The Affiliated Bozhou Hospital of Anhui Medical University, Bozhou, China; ^2^ Department of Respiratory and Critical Care Medicine, The Affiliated Bozhou Hospital of Anhui Medical University, Bozhou, China

**Keywords:** anterior cruciate ligament injury, anterior cruciate ligament reconstruction, knee fibrosis, risk factors, treatment

## Abstract

Anterior cruciate ligament (ACL) injury is a common sports injury, and ACL reconstruction is an effective surgery for this trauma. Most cases gain good recovery after surgery, while some patients may experience knee stiffness, which is characterized by joint fibrosis, leading to reduced joint mobility, pain, and dysfunction. Currently, various research studies have been conducted to unveil the mechanisms underlying this condition, identifying pre-, intra-, and post-operative risk factors, and testify the efficacy of different therapeutic methods against it. In this review, we summarize the current progress regarding the advancements in knee fibrosis after ACL reconstruction. The risk factors associated with knee fibrosis are systematically delineated, accompanied by an evaluation of the efficacy of various treatment modalities for both the prevention and mitigation of fibrosis. Furthermore, recommendations for future research directions are proposed, offering a foundational basis for subsequent investigations.

## 1 Introduction

Anterior cruciate ligament (ACL) injury is a common sports-related knee injury among athletically active people (Chia et al., 2022). Arthroscopic reconstruction of the ACL is the prevalent therapy at present, with generally good recovery and a relatively low complication rate (Hanus and HudÁk, 2020). Still, knee fibrosis, intractable pain, hemarthrosis, fever, deep vein thrombosis, and infection may occur (Hanus and HudÁk, 2020). Knee fibrosis after ACL reconstruction poses a serious problem. According to the literature review, the prevalence of knee fibrosis after ACL reconstruction is 2.0%–35.0% (Eckenrode and Sennett, 2011). Knee fibrosis is characterized by an inflammatory and fibrotic response, which is manifested as a limited range of motion (ROM) and pain, affecting functional recovery (Millett et al., 2001). Knee arthrofibrosis is a joint disorder induced by an overactive inflammatory response. It is characterized by knee pain and decreased range of motion, resulting in impaired joint function. This not only causes great pain and a heavy medical burden for patients but also has a negative impact on the recovery process and long-term prognosis. To improve postoperative outcomes, it is essential to understand the mechanisms, risk factors, and treatment approaches associated with knee fibrosis following ACL reconstruction.

## 2 Pathophysiological mechanisms of knee fibrosis

Knee fibrosis is characterized by the uninhibited deposition of extracellular matrix proteins around the joint, resulting in symptomatic joint stiffness. Fibrosis is the final common pathway of many chronic inflammatory injuries and is a pathological feature of almost all organ diseases (Lee et al., 2022). This article discusses several possible pathological mechanisms, such as inflammatory response, activation and differentiation of fibroblasts, remodeling of the extracellular matrix, and abnormal proliferation of synovial cells in joints (Bayram et al., 2020). In addition, some articles have pointed out that the occurrence of connective tissue fibrosis is multifactorial, including immune cell infiltration caused by tissue damage and the involvement of a series of mediators, such as transforming growth factor-β (TGF-β), bone morphogenetic protein, connective tissue growth factor, and interleukin (Usher et al., 2019; Disser et al., 2023). TGF-β is the pivotal driver of fibrosis, resulting in the activation of fibroblasts and the migration of exogenous cells invading from outside of the tissue. It is a key factor in the regulation of fibroblast proliferation and collagen deposition (Usher et al., 2019). Many of these cells are defined as myofibroblasts, which can produce high levels of alpha-smooth muscle actin and lead to upregulation of collagen synthesis. The excessive activation of immune cells, signaling molecules, and myofibroblasts leads to unresolved post-injury inflammation, which in turn leads to the dysregulation of normal regenerative pathways and formation of fibrous scars (Bayram et al., 2020; Disser et al., 2023). A related report examines the molecular pathological features of human knee fibrosis using RNA sequencing (Jovic et al., 2022). In patients with knee fibrosis, members of the collagen family are commonly expressed as extracellular matrix-related genes, among which COL1A1, COL3A1, and COL6A1 are consistent with fibrosis characteristics (Disser et al., 2023; Morita et al., 2016; Theocharidis et al., 2016; Tao et al., 2018; Samokhin et al., 2018). In addition, integrins are another prominent family in the gene family associated with extracellular matrix organization, and the role of integrins in fibrosis has been confirmed (Disser et al., 2023; Kuivaniemi and Tromp, 2019). Moreover, LOX genes also play a potential role in fibrosis development (Disser et al., 2023; Schnittert et al., 2018). These findings provide new targets for diagnosis and drug therapy.

## 3 Risk factors for knee fibrosis

Knee fibrosis is a multifactorial disease, and its risk factors run through the preoperative, intraoperative, and postoperative periods. Understanding these risk factors can provide guidance for clinical intervention and improve recovery. Personalized treatment and rehabilitation programs are particularly important for patients with multiple risk factors.

### 3.1 Patient characteristics and preoperative risk factors

Studies have identified that factors such as female gender and older age are associated with an increased risk of revision operation after ACL reconstruction due to joint fibrosis. Female patients have a smaller femoral notch than male patients, indicating a structural difference in the joint that may predispose them to arthrofibrosis; older patients are also more prone to chronic injury, which, when combined with degenerative changes, may result in elevated inflammation (Hopper et al., 2024; Haley et al., 2023).

The timing of surgery after ACL injury is suspected to be relevant to the risk of joint stiffness and fibrosis (Freshman et al., 2023) since inflammatory mediators are present in the synovial fluid during the first week after ACL injury (Aman et al., 2024; Kingery et al., 2022; Haslauer et al., 2014). This belief is supported by the evidence that ACL reconstruction performed at least 6 weeks after injury can significantly reduce the risk of surgical intervention for subsequent knee fibrosis (Agarwal et al., 2023). However, this finding was not supported by recent evidence (von Essen et al., 2020). Given these controversial reports, Vermeijden et al. (2023) conducted a systematic review and identified that early surgery is not inferior to delayed surgery regarding knee fibrosis after isolated ACL reconstruction.

The application of anticoagulants is also related to joint fibrosis. Qin et al. found that, compared with patients who did not use thromboprophylaxis, those who took this medication were significantly associated with arthrofibrosis after subsequent surgery (Qin et al., 2021). Thromboprophylaxis results in increased rate of postoperative hematoma and, consequently, inflammatory cytokines within the joint, which may lead to fibrosis. Preoperative knee restriction is a well-established risk factor for arthrofibrosis (Mayr et al., 2004). Therefore, preoperative medication and the limited range of motion should be considered when making surgical plans to reduce the risk of joint fibrosis. In addition, other studies have found that preoperative depression has a negative impact on postoperative pain and functional recovery (García et al., 2024). Patients with preoperative depression have significantly higher pain interference scores and significantly lower physical function scores before and after surgery. At present, many scholars have found that there is a certain relationship between knee joint fibrosis and genetic factors (Skutek et al., 2004; Dagneaux et al., 2020). Comorbidities, including but not limited to type 2 diabetes mellitus, ankylosing spondylitis, and rheumatoid arthritis, are also found to increase the risk of knee fibrosis (Huang et al., 2013; Owen et al., 2021).

### 3.2 Intraoperative risk factors

At present, the autograft options for ACL reconstruction include bone–patellar tendon–bone (BTB), hamstring tendon, and quadriceps tendon. An analysis of 378 patients found that the incidence of knee joint fibrosis with BTB grafts was approximately 10.0%, compared to 1.9% with hamstring tendons and 6.3% with quadriceps tendons (Ouweleen et al., 2021). This phenomenon is suspected to be a consequence of higher collagen content in BTB grafts (Huleatt et al., 2018). Previous studies have suggested a link between graft type and knee fibrosis. Nwachukwu et al. (2011) found that using an autologous patellar tendon was a risk factor for arthrofibrosis after ACL reconstruction (Nwachukwu et al., 2011). Furthermore, Sanders et al. (2017) found that using allografts lowered the likelihood of arthrofibrosis as compared to bone-patellar tendon-bone grafts. Other studies noticed that a femoral tunnel diameter less than 9.25 mm was associated with a reduced risk of joint fibrosis compared to its counterpart in male patients (Haley et al., 2023).

In relation to graft tension, some believe that increasing graft tension creates excessive constraints on the joint and results in loss of movement (Elias et al., 2009). However, studies have shown that although high graft pretension may cause graft wear in the femoral tunnel, it does not lead to complete loss of knee extension (Markolf et al., 1996). Conversely, inadequate graft tension may lead to anterior–posterior laxity, resulting in instability, poor graft healing, and failure (McDermott et al., 2024; Lee et al., 2018; Magit et al., 2007). Increasing the tension of the graft reduces the postoperative loss of tension and mobility due to viscoelasticity. This means that by increasing the tension of the graft, postoperative knee laxity can be reduced. Therefore, there is a relationship between graft tension and knee stiffness, yet there is no clear answer as to whether increasing or decreasing graft tension leads to loss of motion.

In addition, the effect of bone tunnel position and graft placement on fibrosis during ligament reconstruction is important. Placing ACL grafts in anatomical positions can reduce the risk of joint stiffness, while placing ACL grafts in non-anatomical positions may lead to higher rates of fibrosis (Yaru et al., 1992; Tanksley et al., 2017; Romano et al., 1993; Śmigielski et al., 2016; Vignos et al., 2020; Markolf et al., 2002). Multiple studies have found that ACL reconstruction combined with meniscus repair surgery increases the risk of knee fibrosis (Hopper et al., 2024; Haley et al., 2023; Huleatt et al., 2018). Meniscal repair often requires fixation to the joint capsule, which may limit the range of motion of the knee, thus increasing the risk of fibrosis. Moreover, the increase in intra-articular blood loss is also linked to a higher rate of joint fibrosis (Karaaslan et al., 2015).

### 3.3 Postoperative risk factors

Non-standard or excessive postoperative rehabilitation training and postoperative infection may lead to further injury in the joints and increase the risk of fibrosis. Some studies have found that different postoperative weight-bearing protocols (delayed weight-bearing, progressive weight-bearing, and immediate weight-bearing) have different complication rates, among which the delayed weight-bearing protocol has the highest risk of developing stiffness (Morris et al., 2021). Furthermore, reports have pointed out that patients who undergo progressive rehabilitation training after ACL reconstruction surgery have knee function, range of motion, and muscle strength (Grindem et al., 2015; Noyes et al., 2000). The application of a brace can also contribute to the prevention of knee stiffness following ACL reconstruction (Skalsky and McDonald, 2012), while a brace in the hyperextension position for at least 3 weeks was more effective in preserving extension function (Melegati et al., 2003).

## 4 Treatments

**FIGURE 1:**
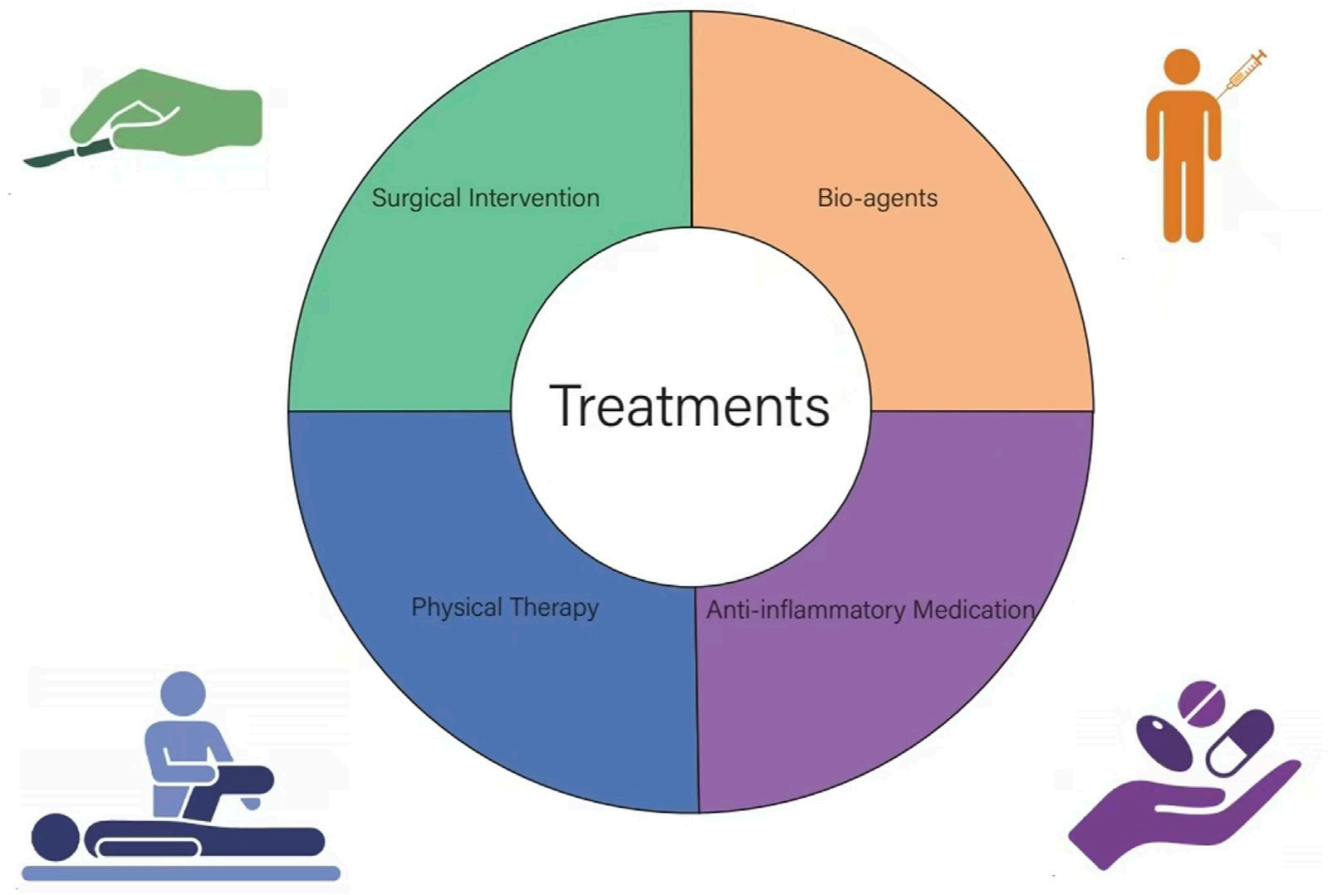
Treatments of knee fibrosis after ACL reconstruction.

Treatments are mainly non-surgical and surgical (Figure 1). Non-surgical treatment includes physical therapy and medication. In severe cases of fibrosis, arthroscopic surgery is required to restore joint mobility. Additionally, postoperative rehabilitation after secondary surgical release is still needed to avoid recurrence.

### 4.1 Non-surgical treatment

Low-level laser therapy (LLLT) and continuous passive motion (CPM) are commonly used physical therapies. Studies have shown that LLLT after ACL reconstruction can reduce the formation of joint contractures by inhibiting inflammation and fibrosis (Kaneguchi et al., 2019). LLLT has anti-inflammatory and anti-fibrotic effects and causes fewer adverse reactions (Kaneguchi et al., 2019; Zhang et al., 2022; Wickenheisser et al., 2019). Moreover, it is a low-cost treatment and is widely used for a wide range of inflammatory and fibrotic diseases (Zhang et al., 2022; Khansa et al., 2016; Soleimanpour et al., 2014). Similarly, CPM treatment can reduce the incidence of knee fibrosis after various knee surgeries (Bram et al., 2019; Haller et al., 2015; Harvey et al., 2010). A recent study using an animal model of ACL rupture showed that immediate CPM therapy has a chondroprotective effect against post-traumatic osteoarthritis (Chang et al., 2017). On the contrary, in two recently published systematic reviews regarding CPM on knee ROM after ACL reconstruction, no evidence is noticed to support the application of this method in the index knee after ACL surgery (Thrush et al., 2018; D'Amore et al., 2021). Therefore, further research is required to evaluate the potential utility of CPM in the long run.

Regarding medications, the main anti-inflammatory drugs used to treat knee fibrosis can be categorized into glucocorticoids and non-steroidal anti-inflammatory drugs (Usher et al., 2019; Liu et al., 2017). The most commonly used non-steroidal drug is aspirin. Aspirin inhibits the development of fibrosis through a variety of mechanisms (Xu et al., 2022; Peng et al., 2023). Aspirin inhibits NF-κB synthesis via IKK receptors and promotes the formation of stable and powerful specialized pro-resolving lipid mediators (SPMs) (Liu et al., 2017). It is possible that aspirin lowers the incidence of fibrosis by decreasing PI3K/AKT/mTOR (phosphorylated phosphatidylinositol 3 kinase, protein kinase B, and mechanistic target of rapamycin) and increasing autophagy (Peng et al., 2023). These mechanisms make aspirin the primary drug currently prescribed for the treatment of fibrosis. Both oral and intra-articular glucocorticoids have advantages and disadvantages in the treatment of joint fibrosis (Barel et al., 2010; Melgert et al., 2001). Oral glucocorticoids can reduce joint inflammation and pain through systemic circulation, but multiple doses are required to maintain the therapeutic effectiveness, which can cause systemic side effects. On the other hand, intra-articular injection can act directly on the inflammatory and fibrotic tissue, improving treatment efficacy and reducing systemic side effects.

By managing the pro-inflammatory and pro-fibrogenic pathways, bio-agents against fibrotic disorders have attracted increasing attention in recent years. Montelukast and Pranlukast are two cytoplasmic leukotriene receptor antagonists mainly used to treat respiratory diseases such as asthma and allergic rhinitis (Wenzel, 1998; Huang and Handel, 2010; Menkü Özdemir et al., 2022; Lynch et al., 1999). In the treatment of arthrofibrosis, these two drugs show therapeutic potential in reducing the postoperative inflammatory response after joint surgery (Chen et al., 2024). Relaxin-2 (RLX-2) is an endogenous anti-fibrotic peptide that is capable of alleviating TGF-β-induced myofibroblast differentiation (Wang et al., 2016; Samuel et al., 2016; Shabanpoor et al., 2012; Sassoli et al., 2013), and thus is used as an anti-fibrotic agent in knee contracture after ACL reconstruction. However, a major obstacle to the clinical translation of RLX is its short half-life (Metra et al., 2019; Khanna et al., 2009; Weiss et al., 2016), which requires further investigations regarding effective delivery modalities. Botulinum toxin type A is currently used as an anti-fibrotic agent for adhesive capsulitis (Blessing et al., 2021; Khenioui et al., 2016; Chen et al., 2011) and is observed to reduce scar formation in animal models of knee fibrosis (Namazi and Torabi, 2007; Gao et al., 2017). Platelet-rich plasma also has potential against joint fibrosis (Araya et al., 2020; Lin et al., 2023). Intra-articular delivery of hyaluronic acid is also a good method for treating knee fibrosis in animal models, while there are few clinical trials testing the efficacy of knee stiffness after ACL reconstruction (Kanazawa et al., 2015; Qu et al., 2023). In addition, vitamin D and angiotensin II receptor antagonists have also been successfully used under different fibrosis conditions and are becoming ideal candidates for joint fibrosis (Jagodzinski and Traut, 2022).

### 4.2 Surgical treatment

Surgical intervention for fibrosis mainly includes manual release under anesthesia (MUA) and arthroscopic lysis of adhesions (LOA). Patients who did not reach a full extension by 3 months postoperatively, defined as lacking 10°, and had a symptomatic difference in the range of motion relative to the unaffected knee were eligible for MUA or LOA. If MUA did not provide a sufficient range of motion, arthroscopy with LOA was recommended instead (Crabtree et al., 2023). MUA is also commonly used to treat knee fibrosis, either alone or in combination with arthroscopy (Crabtree et al., 2023; Baghdadi et al., 2022). For severe fibrosis, soft tissue release via LOA is still the recommended option. By removing the excessive extracellular matrix, LOA can not only relieve the joint movement restriction but also dilute the concentration of intra-articular pro-fibrotic mediators, thus blocking the vicious cycle formed by the ECM (Sanders et al., 2017; Lamba et al., 2023).

Arthroscopic LOA and MUA are safe and effective treatments for the postoperative fibrosis of the knee (Fackler et al., 2022). However, both techniques have complications. These surgical procedures may lead to neurological and vascular disorders, fractures, ligament relaxation, etc (Pivec et al., 2013; Egol et al., 2005; Laskin and Beksac, 2004; Fisher and Shelbourne, 1993). Therefore, careful pre-operative planning is necessary in facilitating knee function after the operation.

In current clinical practice, the prevention of fibrosis development is still challenging. If a physician or surgeon identifies a trend toward knee stiffness, interventions such as physiotherapy regimens and antifibrotic or anti-inflammatory medication can be considered. However, given the lack of evidence-based decision-making, the establishment of a sequential prevention method is still in progress.

Currently, there are studies on the treatment of arthrofibrosis, but reports are still in the basic research stage. In the future, one can consider exploring the mechanism of occurrence and development from the perspectives of molecular biology and genetics, while also searching for new biomarkers and therapeutic targets to facilitate early diagnosis and intervention. In addition, personalized rehabilitation programs and prevention strategies based on specific patient characteristics can be developed to improve efficacy.

## 5 Conclusion

Knee fibrosis after anterior cruciate ligament reconstruction is a complex complication involving multiple risk factors. Early identification and intervention are essential in preventing or treating this condition. Conservative treatment may be useful in the early stages of joint fibrosis, while secondary surgery should be considered in the advanced stage. Determining the appropriate treatment plan requires assessment and decision-making by the physician based on the patient’s specific situation. Future research is still required to explore the biological mechanisms and establish risk models to predict the occurrence of this condition, thereby improving the prognosis of patients after ACL reconstruction.
